# A case of aortico-left ventricular tunnel previously confused with right coronary cusp aneurysm

**DOI:** 10.1186/s44348-024-00008-3

**Published:** 2024-06-12

**Authors:** Yong Beom Cho, Hyun Chung, Gi Beom Kim, Sang Yun Lee, Woong Han Kim

**Affiliations:** 1 Division of Pediatric Cardiology, Department of Pediatrics, Seoul National University Children’s Hospital Seoul National University College of Medicine, 101 Daehak-ro, Jongno-gu, Seoul, 03080 Korea; 2 Department of Pediatrics, Sejong General Hospital, Bucheon, Korea; 3 Department of Thoracic and Cardiovascular Surgery, Seoul National University Hospital, Seoul, Korea

**Fig. 1:**
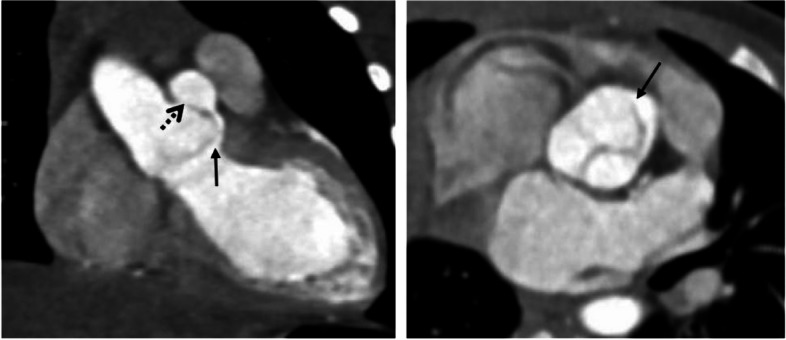
Cardiac computed tomography. Aortico-left ventricular tunnel originating from sinotubular junction (dotted arrow) above right coronary sinus, running lateral side of right coronary cusp, and communicating with left ventricle beneath left–right coronary commissure (bold arrow)

**Fig. 2:**
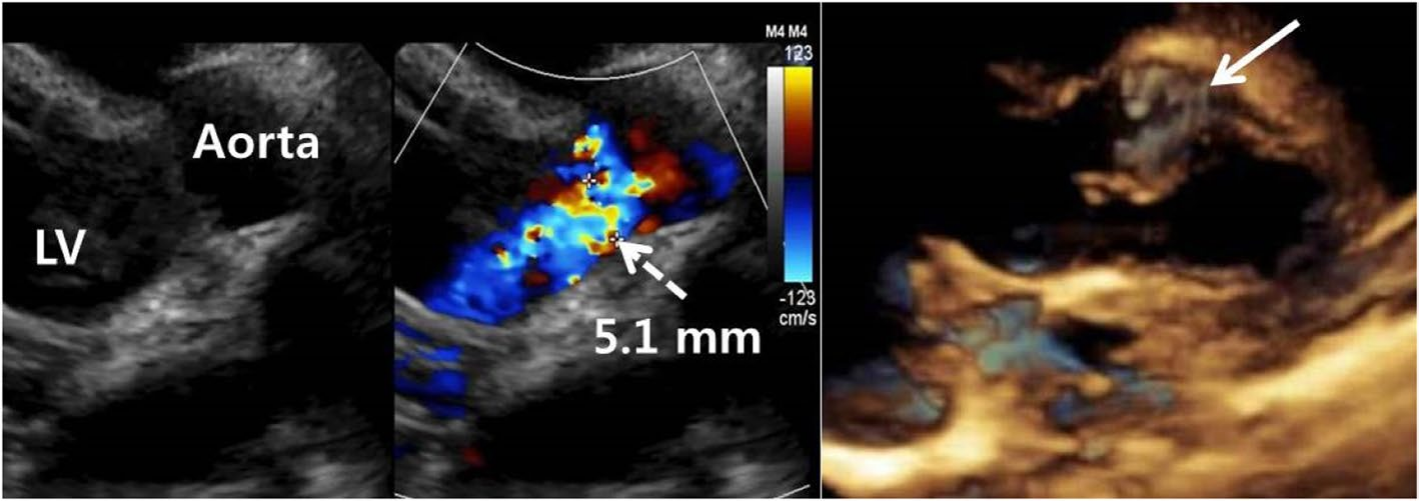
Transthoracic echocardiography. Aortico-left ventricular tunnel (bold arrow) with moderate AR about 5.1 mm color width (dotted arrow). AR: aortic regurgitation, LV: left ventricle

**Fig. 3:**
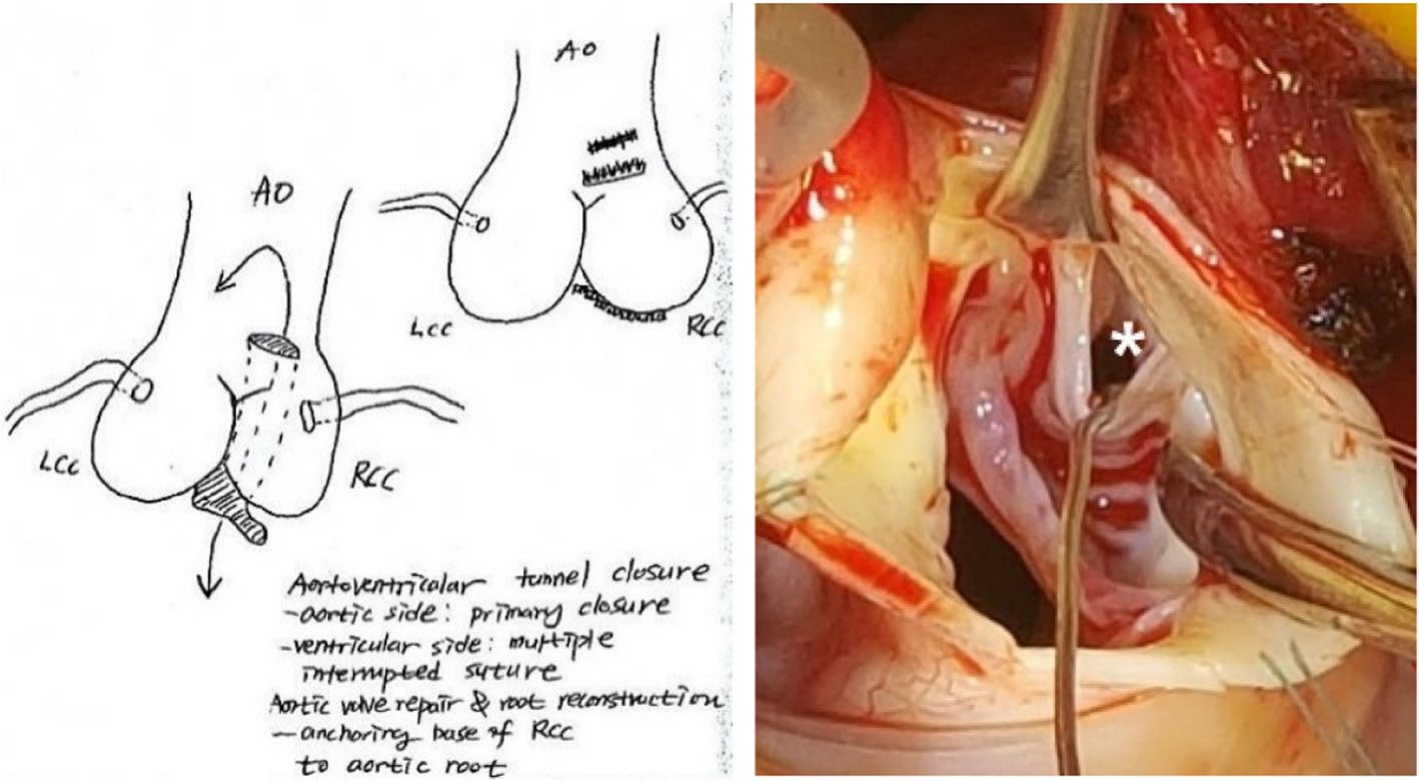
Operative record and field. Direct suture of both ends of aortico-left ventricular tunnel and anchoring base of RCC to aortic root were performed under cardiopulmonary bypass, (asterisk; aortic end). AO: aorta, LCC: left coronary cusp, RCC: right coronary cusp

A 27-day-old neonate was referred for aortic regurgitation (AR) which was detected prenatally and later diagnosed as bicuspid aortic valve with right coronary cusp (RCC) aneurysm by postnatal echocardiography. Because of enlarged left ventricle (LV) with associated AR, enalapril, furosemide and spironolactone were prescribed. Transthoracic echocardiography (TTE) taken one month later revealed mild AR, functional bicuspid aortic valve without aortic stenosis, and borderline LV function (ejection fraction; 54.5%). Because of progressive LV enlargement on TTE at 11 months of age, cardiac computed tomography (CT) was performed for planning surgical correction. Unexpectedly, aortico-left ventricular tunnel (ALVT) originating from sinotubular junction (dotted arrow) above right coronary sinus, running lateral side of RCC, and communicating with LV beneath left–right coronary commissure was found (Fig. 1). Afterward, focused echocardiography could visualize the ALVT (thick arrow) with moderate AR about 5.1 mm color width (Fig. 2). Surgical procedures including direct suture of both ends, and anchoring base of RCC to aortic root were given to the patient (Fig. 3). TTE after the surgery showed trivial AR without residual ALVT. The patient was discharged without any complication.

Now, she is 7 years old without any symptom. Final TTE on Dec. 2022 showed still prominent aortic root with aortic annulus (20.5mm, Z-value: 3.46) and aortic sinus (27.9mm, Z-value: 3.18) which need regular follow-up until adulthood.

This is the first reported case of ALVT in Korea and our case has a typical course of tunnel. This malformation was first described in an infant by Levy et al. in 1963 [1]. The etiology of ALVT is still poor understanding. Although ALVT is relatively common compared to aortico-right ventricular tunnel, ALVT itself is a rare anomaly representing 0.001% of congenitally malformed hearts [2]. ALVT can be diagnosed using echocardiography as the diagnostic investigation of choice, but it can also be diagnosed through CT or magnetic resonance imaging. Antenatal diagnosis by fetal echocardiography is reliable after 18 weeks gestation [3]. Whereas most patients develop symptoms of heart failure during the first year of life, long-term outcomes after surgery are generally good [4]. Therefore, if an infant presents a significant amount of AR with abnormal geometry of aortic root, ALVT should be considered as one of the causes [5]. All patients require lifetime follow-up for aortic valve insufficiency, left ventricular function, and enlargement of the ascending aorta.
